# A Comprehensive Evaluation of Standardized Assessment Tools in the Diagnosis of Fibromyalgia and in the Assessment of Fibromyalgia Severity

**DOI:** 10.1155/2012/653714

**Published:** 2011-10-10

**Authors:** Chad S. Boomershine

**Affiliations:** Boomershine Wellness Centers, Vanderbilt University, 125 Cool Springs Boulevard, Suite 240, Franklin, TN 37067-6475, USA

## Abstract

Standard assessments for fibromyalgia (FM) diagnosis and core FM symptom domains are needed for biomarker development and treatment trials. Diagnostic and symptom assessments are reviewed and recommendations are made for standards. Recommendations for existing assessments include the American College of Rheumatology FM classification criteria using the manual tender point Survey for diagnosis, the brief pain inventory average pain visual analogue scale for pain intensity, the function subscale of the revised fibromyalgia impact questionnaire (FIQR) for physical function, the patient global impression of change and FIQR for overall/global improvement, the hospital anxiety and depression scale depression subscale for depression, the multiple ability self-report questionnaire for cognitive dysfunction, the fatigue severity scale for fatigue, the FIQR for multidimensional function/health-related quality of life, the jenkins sleep scale for sleep disturbance, and the fibromyalgia intensity score for tenderness. Forthcoming assessments including the FIQR for diagnosis, NIH PROMIS, and FIBRO Change scales are discussed.

## 1. Introduction

Fibromyalgia (FM) is one of the most challenging disorders to manage. Treatment advances are needed to improve the care of FM patients. FM is currently a very subjective disorder, and the development of biomarkers could improve care by simplifying FM diagnosis and objectively quantifying symptom severity. Numerous FM biomarkers have been proposed [1]. However, a recent review characterized the current state of FM biomarker development as an “abyss” [1]. Biomarker development has been limited by the lack of universal, “gold standard” definitions for FM clinical diagnosis and symptom severity against which biomarkers can be compared. Similarly, a standard set of assessments for all core FM symptom domains is needed for inclusion in treatment trials to develop better therapies and improve the ability to make efficacy comparisons between treatments. Unfortunately, consensus on standard assessments in research to quantify the severity of FM symptoms is lacking, and recommendations for standard assessments have not been made previously. This paper represents one author's review of the available literature and his recommendations for current and future assessments to clinically diagnose FM and measure the severity of FM symptoms in research to enable development of new therapies and biomarkers. The recommendations made in this paper are intended to be a starting point for discussions in a group such as outcome measures in rheumatology (OMERACT) rather than the final articulation of standards to be used. While none of the assessments discussed herein are perfect, consensus within the FM research community must be reached if timely advances for improving patient care are to be made.

## 2. Recommended FM Clinical Diagnostic  Criteria

FM is a very complex disorder. In addition to widespread pain and tenderness, FM patients also typically suffer from numerous other symptoms that can include, but are not limited to, fatigue, cognitive dysfunction (fibrofog), disturbed (nonrestorative) sleep, depression, anxiety, stiffness, tenderness and functional disability [2]. The number and severity of associated symptoms varies from patient-to-patient, making development of unified diagnostic criteria difficult. While not originally intended for clinical diagnosis, the American College of Rheumatology (ACR) FM classification criteria have been used in the clinic to identify FM patients since their publication in 1990 [3]. These criteria include the presence of widespread pain for at least 3 months and pain upon palpation of at least 11 of 18 tender points with 4 kg/cm^2^ of force. However, performance of the classification criteria in clinical diagnosis is poor, failing to identify almost half of FM patients [4]. 

New ACR FM diagnostic criteria have been proposed to simplify clinical diagnosis by doing away with the need for performing the tender point examination. The new criteria diagnose FM by evaluating the distribution of body pain in combination with symptom severity [5]. While these new criteria have been provisionally accepted by the ACR, final acceptance is awaiting validation studies. However, these new criteria have been criticized for their lack of precision or mechanistic features and complete symptom focus [6–8]. Also, diagnosis via the new criteria is based on a physician's subjective assessment of the extent and severity of the patient's somatic symptoms [9]. Therefore, FM diagnosis using the new criteria is likely to differ from one physician to another. Due to these issues, the new diagnostic criteria cannot be recommended until validation studies are completed.

Though not perfect, the 1990 ACR classification criteria has been used to identify FM patients for inclusion in clinical trials for the past 20 years, and broad consensus exists for its use. At least part of the poor performance of the 1990 ACR criteria in clinical diagnosis is likely due to nonstandard performance of the tender point examination. Performance of the tender point exam can be improved by using the manual tender point Survey (MTPS) method [10]. The MTPS has been shown to reduce variability in performance of the tender point exam and identify FM patients with high sensitivity and specificity [10]. The MTPS consists of standardized components including (1) location of the tender point sites, (2) patient and examiner positioning, (3) order of tender point examination, (4) pressure application technique, and (5) pain severity rating scores in which FM patients rate pain severity upon digital palpation of each tender point on a verbal 11-point numerical rating scale (NRS) from 0 (no pain) to 10 (worst pain), with a pain severity score of at least 2 required to count a tender point as positive. Until the new FM diagnostic criteria are validated, the 1990 ACR fibromyalgia classification criteria utilizing the MTPS are recommended for the diagnosis of FM patients in research studies (Table 1).

## 3. Recommended FM Core Symptom Domains

As previously discussed, FM is a complex disorder with numerous symptoms occurring along with widespread pain and tenderness [2]. Since effective FM management requires a treatment regimen that addresses not only pain but all associated FM symptoms [11], multiple biomarkers will likely be necessary to evaluate all FM symptom domains. While phase-three FM treatment trials have evaluated multiple symptom domains (Table 2), the symptoms assessed across trials have been inconsistent. This is likely because the US Food and Drug Administration (FDA) has made the improvement of pain the primary consideration for approval of FM medications, and a required core set of symptom domains for evaluation in FM treatment trials does not exist. Discrepancies in symptoms assessed by treatment trials can lead to bias, since researchers tend to only evaluate symptoms likely to be improved by the treatment under investigation and ignore symptoms likely to be made worse. A required core set of symptom domains are needed so that a comprehensive set of FM biomarkers can be developed and bias in treatment trials can be reduced.

Recommendations for a core set of FM symptom domains to be assessed in treatment trials have been made previously (Table 1) [12]. These recommendations arose from a Delphi exercise of FM patients and expert clinicians to determine symptoms that should be evaluated in all treatment trials [2]. While it may be advisable for additional symptoms to be evaluated in some circumstances (e.g., anxiety for an antidepressant trial), a core set of 9 symptom domains were recommended for assessment including pain intensity, physical function, patient global impression of change, cognitive dysfunction (fibrofog), fatigue, multidimensional function/health-related quality of life (HRQoL), sleep disturbance, tenderness and depression (Table 1). Since consensus for these core domains exists, it is recommended that biomarkers be developed to evaluate these 9 symptom domains.

## 4. No Recommended Assessments for FM Core Symptom Domains Currently Exist

While recommendations for core symptom domains exist, there are no accepted standards for assessments to evaluate these domains. Standard measures are needed against which biomarkers can be compared for development and to allow direct comparisons between treatment trial results. Published work to develop a consensus set of FM outcome measures has been limited to two papers [13, 14]. The work by Choy et al. pooled data from 10 fibromyalgia treatment trials to determine the construct validity of questionnaires that have been used to assess change in a number of FM symptom domains including pain, patient global, fatigue, multidimensional function (which included HRQoL), sleep, depression, physical function, tenderness, fibrofog, anxiety, and stiffness [13]. The authors did not compare questionnaires to one another, and no specific recommendations for assessments were made. Instead, pooled psychometric properties of questionnaires for each symptom domain were assessed. Support was found for construct validity of self-report questionnaires for pain, fatigue, depression, physical function, and multidimensional function. However, support was not found for use of full-length sleep questionnaires (including the medical outcomes studies (MOS) sleep scale). The lack of support for sleep questionnaires was because many items assessed sleep problems such as snoring and shortness of breath that are not relevant to most FM patients. The authors recommended either using the sleep disturbance subscale of the MOS sleep scale in isolation or developing a new FM-specific sleep questionnaire for use in clinical trials. Validity could also not be determined for fibrofog, anxiety, or stiffness assessments because these domains were measured by only one instrument in the trials (the multiple abilities self-report questionnaire (MASQ), anxiety subscale of the hospital anxiety and depression scale (HADS), and stiffness subscale of the fibromyalgia impact questionnaire (FIQ), resp.). 

The work by Carville and Choy reviewed 185 FM trials to identify assessment instruments for core FM domains that were sensitive to change for both pharmacologic and nonpharmacologic treatments [14]. The authors only compared the 5 assessments that were most often used for each core symptom domain, significantly limiting the questionnaires that were evaluated since concordance of assessments across trials was low. Across all domains including pain, patient global, fatigue, sleep, and anxiety, visual analogue scales (VASs) were found to be sensitive to change. For pain, the FIQ, tender point count, and pressure point threshold were insensitive to change. While evaluation of depression questionnaires was limited due to the use of multiple different assessments across trials and the exclusion of patients with significant depression from many trials, the Hamilton and Center for Epidemiologic Studies Depression scales were found to be more sensitive to change than the Beck depression inventory (BDI). All anxiety scales analyzed were insensitive to change in pharmacologic trials including the Beck anxiety inventory, state trait anxiety index (STAI) and the FIQ anxiety subscale. For fatigue, Likert scales showed good sensitivity, but the FIQ fatigue subscale did not. For health-related quality of life, the physical component of the short form36 was sensitive to change in pharmacologic studies, but the mental component score was not. For sleep, the FIQ sleep item was sensitive to change in nonpharmacologic trials but not in pharmacologic trials. For evaluation of multidimensional function, the global FIQ score was moderately sensitive to change in both pharmacologic and nonpharmacologic trials. While the analyses in this work are instructive, no specific recommendations for assessment measures were made.

## 5. Recommended Pain Intensity Assessment

The initiative on methods, measurement, and pain assessment in clinical trials (IMMPACT) has provided recommendations for questionnaires to be used in interpreting the clinical importance of therapeutic outcomes in clinical trials of chronic pain treatments that may be applicable to FM [15]. IMMPACT recommends assessment of four core chronic pain outcome domains including (1) pain intensity, (2) physical functioning, (3) emotional functioning, and (4) participant ratings of overall improvement. A 0 to 10 NRS was recommended to assess pain intensity, but no specific recommendations for the wording of instructions or anchors of the NRS were made. To ensure uniformity across FM trials, I recommend wording from the average pain NRS of the brief pain inventory (BPI) to be used which asks “please rate your pain by circling the one number that best describes your pain on average” and has anchors that vary from “no pain” to “pain as bad as you can imagine” since this wording can help limit recall bias and reduce ceiling effects commonly seen in FM patient trials [16].

## 6. Recommended Physical Functioning  Assessment

IMMPACT recommends the multidimensional pain inventory (MPI) [17] and the interference scale of the (BPI) [16] for assessment of physical functioning unless a well-validated disease-specific measure is available. Since the FIQ physical functioning subscale is a well-validated FM-specific measure that has been used to assess physical functioning in all FM clinical trials (Table 2) [18], it is reasonable to use it to assess physical functioning. However, the FIQ physical functioning subscale has been criticized for its gender bias, nonlinearity, nonunidimensionality, and systematic underestimation of functional impairment by inclusion of infrequently performed activities [19]. The FIQ was recently revised as the FIQR to address some of these criticisms and allow for computerized administration [20]. Therefore, the FIQR physical functioning subscale is recommended for assessment of physical function in FM patients.

## 7. Recommended Overall/Global Improvement Assessments

The patient global impression of change scale (PGIC) is IMMPACT recommended for evaluating participant ratings of overall improvement in pain treatment trials [15]. The PGIC uses a 7-point Likert scale that varies from 1 “very much improved” to 7 “very much worse” to quantify patient global response to treatment [21]. The PGIC is a standard assessment in clinical trials, and various forms of the PGIC have been used in all FM treatment trials (Table 2). A specific form of the PGIC has been linguistically validated into 12 languages to allow for use in FM research worldwide [22], and this form of the PGIC is recommended to assess overall/global improvement in combination with the FIQR (Table 1).

## 8. Recommended Depression Assessment

The beck depression inventory (BDI) is the IMMPACT recommended depression assessment for pain treatment trials [15]. The BDI was recommended based on its excellent psychometric properties, extensive use in pain clinical research, and responsiveness to change in pain clinical trials. The BDI consists of 21 multiple choice items, with each item answered by choosing which of four statements best describes the way the patient feels [23]. The BDI was designed to assess the severity of current depressive symptoms, with scores ranging from 0 to 63 with higher scores indicating more severe depression. The original BDI evaluated symptoms over the past week, but this time frame was increased to two weeks for the current version of the BDI, the BDI-II, for consistency with *Diagnostic and Statistical Manual on Mental Disorders *(*DSM-IV*) criteria for major depressive disorder [24]. The BDI-II was also modified to include assessments for all nine *DSM-IV *criteria (the original BDI only met six of the nine criteria). The BDI-II has been linguistically validated into 12 languages for use in international FM treatment trials [22]. Normative data on the BDI and BDI-II are available.

While the BDI is recommended for use in pain treatment trials and has been used in FM trials (Table 2), it is not recommended to quantify depression severity in FM patients due to its lack of unidimensionality. The BDI does not specify patients answer on the basis of their depressive symptoms but to comment on how they “…have been feeling.” Only one of the 21 BDI-II items specifically addresses feelings of sadness, the rest of the items query general physical and mental concepts not specific for depression including tiredness/fatigue, concentration difficulty, sleep changes, and loss of energy. Given the myriad of coexisting FM symptoms, it is likely the BDI functions as more of a general measure of symptomatology in FM patients rather than a specific measure of depression symptom severity. This view is supported by a study comparing the BDI to a computerized diagnostic interview schedule (C-DIS) for diagnosing major depressive disorder (MDD) in FM patients [25]. The C-DIS is a reliable method for identifying individuals with MDD [26, 27]. This study found that while the C-DIS detected MDD in 22% of FM patients, the BDI estimate was much higher at 55%. The authors felt that the BDI overestimated the presence of MDD because numerous BDI items evaluate nondepressive symptoms that are part of the FM syndrome process and that the BDI lacked utility as a unidimensional measure of depression in FM patients. Factor analyses of the BDI have identified three dimensions, including negative attitudes toward self (also termed general depression), performance impairment and somatic concerns [28, 29]. The BDI-II has also been shown to be multidimensional, composed of two first-order factors representing cognitive and noncognitive symptoms [24, 30]. It is very important that assessments used in treatment trials and to develop biomarkers be unidimensional to ensure specificity. Given the lack of specificity of the BDI for assessing depression symptoms, it cannot be recommended to quantify depression severity in FM trials.

The HADS depression subscale has been used previously to evaluate change in depressive symptoms in FM treatment trials (Table 2). The HADS was originally developed to quantify the severity of anxiety and depressive symptoms in nonpsychiatric hospital clinic patients [31]. To prevent somatic disorders common in these patients from falsely affecting scores, symptoms of anxiety and depression that relate to physical disorders were excluded to ensure unidimensionality of the anxiety and depression subscales that have been confirmed by factor analysis in multiple populations [32]. The HADS includes 7 items each to assess anxiety and depressive symptoms (the HADS-A and HADS-D, resp.), with each item answered on a four-point (0 to 3) scale so that possible scores range from 0 to 21 for both anxiety and depressive symptoms with higher scores indicating more severe symptoms. The HADS has been translated into a number of languages allowing for its use in international trials.

While the HADS-D has not been specifically validated in FM patients, its validity to quantify depression symptoms has been shown in a variety of patient populations including somatic, psychiatric, and primary care patients and in the general population [32]. The HADS-D also has proven validity in its ability to reflect change in depression severity in treatment trials [33]. An analysis of data from three pregabalin FM trials supports diagnostic validity of the HADS by showing that, using a standard cutoff score of ≥11 on the HADS-D to identify patients with MDD, 27% of enrolled patients in the trials had MDD, a percentage consistent with previously reported MDD rates in FM patients when gold-standard diagnostic assessments are done [34]. Similarly, 38% of patients in the FM pregabalin trials were found to have an anxiety disorder using the HADS-A, consistent with previously published rates in FM patients [34, 35]. Based on this, the HADS-D is recommended for assessment of depressive symptoms in FM patients (Table 1).

## 9. Cognitive Dysfunction Assessments

Core FM domains that lack published recommendations for self-report questionnaires include cognitive dysfunction, fatigue, multidimensional function/HRQoL, sleep, and tenderness. While numerous assessments exist for each domain in the literature, most were developed for use in patient populations other than FM. This is significant, since concepts and psychometric properties of a measure may not hold across patient populations. While cognitive dysfunction is a significant problem for many FM patients, assessment in large clinical trials has been hindered by the time and expertise required to perform the complex neurocognitive tests that can quantify cognitive dysfunction in FM [36]. The multiple ability self-report questionnaire (MASQ) is the only self-report questionnaire that has been used in FM clinical trials to assess cognitive dysfunction [37]. The MASQ is a self-report measure comprising items to assess five cognitive domains including language, visuoperception, verbal memory, visual memory, and attention. While originally written in English, the MASQ has been translated and linguistically validated into 12 different languages to facilitate its use in international FM studies [22]. For this reason, the MASQ is recommended for assessment of cognitive dysfunction in fibromyalgia trials. However, it is hoped that computerized batteries of neurocognitive tests will soon become available to provide more objective measures of cognitive function for use in clinical trials [36] since self-assessment is poorly correlated with objective measures of cognitive function and has poor discriminating ability for patients with mild cognitive impairment [38].

## 10. Fatigue Assessments

Numerous self-report questionnaires have been used to assess fatigue severity in FM treatment trials including the multidimensional fatigue index (MFI) [39], the multidimensional assessment of fatigue (MAF) [40], and the fatigue severity scale (FSS) [41]. While none of the fatigue questionnaires were developed in FM patients, the MFI was developed in chronic fatigue syndrome patients who have fatigue symptoms similar to those seen in FM. The MAF was developed in rheumatoid arthritis patients, while the FSS was developed in patients with multiple sclerosis and systemic lupus erythematosus. All three scales are available in numerous languages, allowing for their use in international FM studies.

The MFI is a 20-item scale that covers 5 dimensions: general fatigue, physical fatigue, mental fatigue, reduced motivation and reduced activity. Four items in each subscale are answered on a 5-point Likert scale with item scores summed to yield subscale scores and a total score that varies between 20 and 100 with higher scores indicating more severe fatigue symptoms. The MFI is reliable and valid in FM patients [42], and it has been linguistically validated into 12 languages to enable use in international FM studies [22]. Normative data for the MFI is available for multiple subgroups including the general population [43]. 

The MAF is a 16-item scale (14, 10-point NRSs and 2, 4-point Likert scales) that measures 4 dimensions of self-reported fatigue: severity (2 items), distress (1 item), timing (2 items; how often fatigue occurs and change in fatigue over the past week), and degree of interference with activities of daily living (11 items including household chores, cooking, bathing, dressing, working, socializing, sex, leisure, shopping/errands, walking, and exercise other than walking) [40]. The global fatigue index (GFI), a measure of global fatigue severity derived from 15 of the MAF items, has a scoring range that varies from 7.5 to 50 with higher scores indicating more severe fatigue. While the GFI technically varies from 1 to 50, patients can only score a 1 if they have no fatigue; the lowest possible score for a patient with fatigue is 7.5. The MAF and a user's guide are freely available from the developer's website (http://www.son.washington.edu/research/maf/). The MAF is available in 25 different language versions for various regions of the world, allowing for use in international research. While no published validation studies of the MAF in FM patients exist, a study of the MAF in 663 FM patients presented at EULAR in 2002 supports use of the MAF in the assessment of FM patients [44]. 

The FSS is a 9-item unidimensional measure of fatigue that is the most often used fatigue-specific scale in chronic diseases [41, 45]. The FSS measures fatigue by quantifying the impact of fatigue on specific types of functioning rather than the intensity of fatigue-related symptoms [46]. Each item is scored on a 7-point NRS, and the FSS score is derived by averaging all items to yield a score from 1 to 7 with higher scores indicating more severe fatigue symptoms. The FSS is freely available in English from the original paper [41] and has been translated into multiple languages. Validity and reliability of the FSS in FM patients has been demonstrated [47].

While all 3 scales were designed to measure fatigue symptoms, they differ considerably in their composition and foci. While the MFI is the most comprehensive of the 3 fatigue scales, questions have been raised about its dimensional structure, and it appears that MFI may only discriminate between two fatigue dimensions [48]. Also, the MFI does not specify patients' answer on the basis of their fatigue symptoms but to comment on how they “…have been feeling lately.” Only two of the 20 MFI items specifically address feelings of tiredness, the rest of the items query general physical and mental concepts not specific to fatigue. Given the myriad of FM symptoms that contribute to physical and mental disability, it is likely that the MFI functions as more of a general symptom measure than a specific measure of fatigue in FM patients. On the basis of these limitations, the MFI cannot be recommended to quantify fatigue severity in FM patients. 

In contrast to the MFI, both the MAF and the FSS instruct patients to answer questions based on their fatigue symptoms, and every question specifically asks about fatigue, making the MAF and FSS more specific fatigue measures than the MFI. The MAF and FSS have both been recommended for the measurement of fatigue in chronic illnesses based on their good psychometric properties and demonstrated ability to detect change over time [49]. However, a comparison of fatigue measures concluded that the FSS had the most robust psychometric properties of 19 reviewed fatigue measures, including both the MAF and the MFI, and had the best ability to act as an outcome measure sensitive to change with treatment [49]. 

The FSS is the recommended fatigue severity measure in multiple disorders with associated fatigue including systemic lupus erythematosus [50] and Parkinson's disease [51]. The FSS is also easier to score than the MFA and is shorter. For this reason, the FSS is the recommended fatigue assessment for use in FM patients.

## 11. Multidimensional Function/HRQoL Assessments

The FIQ is recommended as a primary efficacy endpoint measure in FM clinical trials [52] and is the standard assessment measure for multidimensional function/HRQoL in FM patients, having been cited in over 300 papers and translated into 14 languages. The FIQ is a 20-item self-report questionnaire that quantifies global FM disease severity by measuring the degree to which FM interferes with a patient's life over the past week [18]. The FIQ is divided into 11 items to assess physical function, 2 “day-of-the-week” items to quantify the number of days patients “felt good” or “missed work,” and 7 VASs to assess symptoms of fatigue, sleep quality, depression, anxiety, stiffness, pain, and work disability. The FIQ has been translated into numerous languages enabling its use internationally [22].

While the FIQ is universally used in FM trials, problems with the questionnaire exist. FIQ scoring is complex. The 11 physical function items are each scored on a 4-point Likert scale ranging from “always” (score of 0) to “never” (score of 3). Scores on the physical function items are summed, divided by the number of questions answered, and then multiplied by 3.33 to yield a 0-to-10 composite physical function score. The “felt good” “day-of-the-week” item is reverse scored (to obtain the number of days patients felt bad), and the result is multiplied by 1.43 to yield a 0-to-10 score. The “missed work” “day-of-the-week” item is derived by multiplying the number of days by 1.43 to yield a 0-to-10 score. The VASs are scored by measuring the distance from the beginning of the line to the patient's mark in centimeters. FIQ global scores are derived by summing the 0-to-10 composite physical function, “day-of-the-week,” and VAS item scores to yield a 0-to-100 score with higher scores indicating more severe FM. In order to maintain a maximum possible score of 100, an “equalization calculation” is used if patients did not answer all 10 sections by multiplying the global score by 10 and dividing by the number of sections answered. Content problems in the FIQ have also been raised [20]. The physical function items were originally intended for women living in affluent countries and assumed possession of an automobile, vacuum cleaner, and washing machine. Also, questions now considered relevant to FM symptomatology including cognitive dysfunction, tenderness, balance problems, and environmental sensitivity were not included in the original FIQ. Finally, the original FIQ was developed as a pen-and-paper questionnaire and is incompatible with computer administration. 

To address shortcomings of the original FIQ, the authors have published a revised FIQ, the FIQR [20]. The FIQR uses 21 NRS questions to evaluate the same three domains as the FIQ (physical function, overall impact, and symptoms) but differs from the FIQ by having modified physical function questions and the inclusion of questions on memory, tenderness, balance, and environmental sensitivity. The NRS structure of the FIQR questions simplify scoring and calculation of the subset domain and global scores. As in the FIQ, the FIQR yields a 0-to-100 score with higher scores indicating more severe FM. The FIQR has comparable scoring characteristics to the original FIQ, making comparison of results between the FIQ and the FIQR possible. The FIQR has the added functionality of computer-based administration, and there is a disease-neutral version of the FIQR, the SIQR, that can be used in population studies to identify and study FM patients who have not been previously diagnosed [53]. For these reasons, the FIQR is the recommended multidimensional function/HRQoL assessment for use in FM patients.

## 12. Sleep Assessments

Sleep problems are almost universal in FM, occurring in 95% of patients [2]. Since disturbed sleep can worsen numerous other FM symptoms including pain, depression, and physical disability [2], accurately assessing sleep dysfunction is vital. Three sleep assessments have been used in FM trials (Table 2); the MOS sleep scale [54], the functional outcomes of sleep questionnaire (FOSQ) [55], and the jenkins sleep scale (JSS) [56]. All three scales are available in numerous languages, allowing for their use in international FM studies.

The MOS Sleep Scale is a 12-item questionnaire designed to evaluate key sleep domains by assessing sleep latency (2 items), duration, quality (4 items), snoring, awakening short of breath or with headache, and daytime somnolence (3 items) over the past month [54] or week [57]. All items are answered on a 6-point Likert scale from 1 = “all of the time” to 6 = “none of the time” except for the time to sleep and number of hours of sleep questions which are answered in minutes and hours, respectively. Seven subscales are derived to evaluate sleep disturbance, snoring, shortness of breath or headache, adequacy, somnolence, quantity, and optimal sleep. A sleep problems index can also be calculated from 9 of the items to generate a 0 to 100 score that quantifies overall sleep problems with higher scores indicating worse sleep problems. As previously discussed, an analysis of FM assessment measures showed the MOS sleep scale lacked construct validity to assess change in sleep symptoms in FM treatment trials [13]. This was because the MOS Sleep Scale evaluates numerous problems, such as snoring and shortness of breath, that are not relevant to many FM patients. The analysis concluded that the sleep disturbance subscale may be useful in isolation, but the MOS sleep scale as a whole was not recommended for use in FM patients. Another evaluation of the MOS sleep scale provided some evidence for content validity in FM, but modifications to the scale were recommended to improve the psychometric properties and relevance in FM patients [58]. Based on these analyses and the availability of other sleep questionnaires, the MOS sleep scale is not recommended for quantifying the severity of sleep symptoms in FM patients. 

The FOSQ is a 30-item self-report questionnaire designed to measure the impact of daytime sleepiness on activities of daily living in people with sleep disorders using functional status categories including general productivity, activity level, vigilance, social outcomes, and intimacy and sexual relationships [55]. Each item queries level of difficulty with an activity due to being “sleepy or tired” and is answered from 1 = “yes, extreme (difficulty)” to 4 = “no (difficulty).” FOSQ scores range from 5 to 20 with lower values indicating worse functioning. The FOSQ was developed in patients with disorders of excessive sleepiness, primarily obstructive sleep apnea, recruited from university sleep disorder clinics [55]. The FOSQ has never been validated in FM patients. This is significant, as the FOSQ instructions state that “when the words “sleepy” or “tired” are used, it describes the feeling that you cannot keep your eyes open, your head is droopy, you want to nod off, or you feel the urge to nap. These words do not refer to the tired or fatigued feeling you may have after you exercised.” FM patients commonly have severe worsening of fatigue after exercise not seen in typical patients that is likely to significantly interfere with their ability to correctly complete the questionnaire. For this reason, the FOSQ cannot be recommended until validation and reliability studies are completed in FM patients. 

The JSS is a 4-item self-report questionnaire designed to measure how often a subject has experienced sleep problems in the past month [56]. JSS items evaluate trouble falling asleep, staying asleep, waking up several times, and awakening unrefreshed with each item scored on a 5-point Likert scale from 0 = “not at all” to 5 = “22–31 days.” Scores vary from 0 to 20 with higher scores indicating more frequent sleep problems. The JSS has been studied in FM patients and found to be valid, reliable, and able to detect change after treatment [59]. Of the three sleep questionnaires that have been used in phase III FM trials, the JSS is recommended for assessment of sleep symptoms in FM patients (Table 1). However, the JSS has been criticized for possible high-recall bias because it requires patients to rate frequency over the past month. To limit recall bias, an alternative scoring scheme has been proposed and provisionally validated in FM patients by scoring each item either “not at all,” “less than 1/2 the time,” or “greater than 1/2 the time” [59]. However, further testing of this alternate scoring scheme is needed before it can be recommended for use in assessing FM patients.

## 13. Tenderness Assessments

Tenderness is a defining feature of FM patients, defined by the 1990 ACR criteria as pain upon palpation at standard tender point sites with 4 kg/cm^2^ of force [3]. However, decreases in the number of tender points have been shown to correlate poorly with patient improvement in FM treatment trials [52, 60]. This is likely because tender point counts do not specifically measure tenderness but are a more general measure of distress influenced by cognitive and emotional aspects of pain perception [61, 62]. 

Change in the severity of pain at tender point sites has been shown to be a better measure of tenderness than change in the number of tender points. The manual tender point survey (MTPS) is a standardized approach to performing the tender point exam in which FM patients rate pain severity upon digital palpation of each tender point on a verbal 11 point NRS [10]. Pain severity ratings from the 18 tender points are averaged to yield a Fibromyalgia Intensity Score (FIS) that varies from 0 to 10 with higher scores indicating more severe tenderness. The MTPS/FIS has been used in a pregabalin FM treatment trial, and decreases in FIS scores with treatment were seen [63]. The tender point index (TPI) is a similar tenderness measure to the FIS that supports its use [3]. For the TPI, patients are asked to rate pain on a 0 to 4 scale upon digital palpation of each of the standard 18 FM tender points, and pain scores for all tender points are summed to yield a 0 to 72 score with higher scores indicating more severe tenderness. Significant decreases in TPI scores with active treatment compared to placebo were demonstrated in a small milnacipran FM trial [64], but no other trials have used the TPI. 

While newer assessments currently under development may prove superior for evaluating tenderness, such as the multiple random staircase-evoked pain measure [60], they remain unproven. The MTPS/FIS is the currently recommended tenderness assessment for FM trials since it is standardized, can be performed with minimal training, and does not require specialized equipment.

## 14. Recommended Evaluations and the PROMIS of Future Assessments

As previously discussed, it is currently challenging to compare results across different treatment trials since different measurement tools are commonly used that often have noncomparable or noncombinable scores. This limitation of clinical trials is well known and not exclusive to FM. Two routes have been taken to solve this problem: (1) require a standard set of existing assessment tools to be used in treatment trials or (2) develop a new set of assessment tools for use in clinical trials. While groups like IMMPACT have taken the first route and made recommendations for the use of existing assessment tools [15], these recommendations have typically not been followed because regulatory bodies like the US Food and Drug Administration have not made them mandatory. This is likely due to the fact that, as we have seen in the case of FM, assessments that work well for one condition do not work well for others. Because of this, the FDA would need to develop a different set of recommended assessments for use in treatment trials of every condition.

The US National Institutes of Health roadmap project Patient-Reported Outcomes and Measurement Information System (PROMIS) has taken the second route to address the need for uniform assessment measures in clinical trials. PROMIS is a 5-year cooperative group program of research designed to develop, validate, and standardize item banks to measure patient-reported outcomes relevant across common medical conditions [65]. The PROMIS network is working to combine items from the best of all current patient self-report questionnaires to create a set of standard symptom severity assessments for use in clinical trials. In addition to improving the ability to compare results from one trial to another, use of PROMIS is expected to reduce the sample size requirements of trials needed to demonstrate minimal clinically important differences by 20% to 50%. PROMIS also has great potential in clinical practice to rapidly and reliably assess response to interventions and to inform treatment decisions. PROMIS is particularly well suited for use in FM treatment trials, as assessments have been developed for all core FM symptom domains [66], and field testing of PROMIS item banks in >3500 FM patients is nearing completion (private communication with David A. Williams, University of Michigan, Ann Arbor, Mh, USA). Assuming PROMIS item banks are shown to be valid and reliable in FM patients, they will be the recommended assessment standard for core FM symptom domains in addition to the FIQR and PGIC for global improvement.

While PROMIS is being developed, the athens insomnia scale (AIS) should be studied for use in assessing sleep disturbance in FM patients [67]. The AIS is a recently developed 8-item self-report sleep questionnaire based on ICD-10 insomnia diagnostic criteria that has been recommended for use in therapeutic trials based on its superior feasibility, validity, and psychometric properties compared to 44 other sleep questionnaires including those discussed above [68]. The AIS cannot only quantify the severity of sleep problems but also be used to diagnose patients with insomnia [67]. The AIS has a 5-item subscale, the AIS-5, that is a unidimensional measure of nighttime sleep problems similar to the JSS, and scores on the two scales are highly correlated [67]. However, the AIS is superior to the JSS since it also has 3 items that measure the severity of daytime symptoms related to poor sleep that are lacking in the JSS, and assessing the severity of daytime symptoms is important to evaluate in patients with sleep problems. However, recommendation of the AIS must await validation and reliability testing in FM patients.

High ceiling effects are typically seen when assessments are used to quantify symptom severity in FM patients. As an example, the ceiling effect on the JSS total score was found to be 27% in an FM treatment trial [59]. Assessments with high ceiling effects are problematic because they limit the ability to adequately evaluate patients with severe symptoms and measure symptom worsening (e.g., an FM patient with a maximal JSS score at baseline is treated and, even though their sleep symptoms worsen, the JSS score remains unchanged from baseline which is wrongly interpreted as no change in symptom severity). Patient impression of change scales have been shown to be superior to other questionnaire types in evaluating change in patients with severe symptoms [69]. The FIBRO Change Scale have been proposed as a way to use patient impression of change scales to better evaluate change in FM patient symptoms in response to therapy [70]. The FIBRO change scale includes seven patient impressions of change scales to assess key FM symptoms including fatigue, fibrofog, sleep dysfunction, depression, anxiety, stiffness, and pain each answered on a 0 to 10 scale from 0 = “very much improved,” 5 = “no change” and 10 = “very much worse.” A similar scale could be developed incorporating patient impression of change items for all 9 core FM symptom domains that could reduce the problem of questionnaire floor and ceiling effects in biomarker development and therapeutic efficacy evaluations. However, such a scale will require validation studies in FM patients before it can be recommended.

## 15. Conclusions

Objective biomarkers and new treatments are needed to improve the diagnosis and management of FM. However, research standards for FM clinical diagnosis and core FM symptom domain assessments are needed to enable development of biomarkers and new treatments. Since standards for FM diagnosis and symptom assessment do not exist, these recommendations are intended as a starting point for discussions that will lead to the development of standards. While none of the assessments discussed herein are perfect, consensus within the FM research community must be reached if timely advances for improving patient care are to be made. The 1990 ACR FM classification criteria performed using the manual tender point survey (MTPS) standardized method incorporating the fibromyalgia intensity score (FIS) is the recommended method for clinical diagnosis since the 1990 criteria are well established, and the method can be performed without any specialized equipment. Recommended assessments for core FM symptom domains include the brief pain inventory average pain visual analogue scale for pain intensity, the physical function subscale from the revised fibromyalgia impact questionnaire (FIQR) for physical function, the patient global impression of change (PGIC) and FIQR for overall/global improvement, the depression subscale of the hospital anxiety and depression scale (HADS-D) for depression, the multiple ability self-report questionnaire (MASQ) for cognitive dysfunction, the fatigue severity scale (FSS) for fatigue, the FIQR for multidimensional function/health-related quality of life, the Jenkins Sleep Scale (JSS) for sleep disturbance, and the MTPS-FIS for tenderness. It is hoped that these recommendations will provide an impetus for the development of universally accepted standards of FM clinical diagnosis and symptom domain assessments that can provide the foundation for the development of objective FM biomarkers and new more effective treatment regimens.

## Figures and Tables

**Table 1:** Recommended assessments for fibromyalgia diagnosis and core symptom domains.

Symptom domain	Assessment
Diagnosis	1990 ACR fibromyalgia classification criteria utilizing the MTPS
Pain intensity	NRS from question 5 of the BPI
Physical functioning	FIQR physical functioning subscale
Overall/Global improvement	PGIC and FIQR global score
Depression	HADS-D
Cognitive dysfunction	MASQ
Fatigue	FSS
Multidimensional function/health-related quality of life	FIQR
Sleep disturbance	JSS
Tenderness	MTPS-FIS

ACR: american college of rheumatology, BPI: brief pain inventory, FIQR: revised fibromyalgia impact questionnaire, FIS: fibromyalgia intensity score, FSS: fatigue severity scale, HADS-D: depression subscale of hospital anxiety and depression scale, JSS: jenkins sleep scale, MASQ: multiple abilities self-report questionnaire, MTPS: manual tender point survey, NRS: numeric rating scale, PGIC: patient global impression of change.

**Table 2:** Assessments used to evaluate fibromyalgia symptom severity in medication trials.

Symptom	Medication used in treatment trials			
	Pregabalin	Duloxetine	Milnacipran	Sodium oxybate
Fatigue	MAF	MFI	MFI	Fatigue VAS
Fibrofog	—	—	MASQ	—
Sleep	MOS-Sleep	—	JSS, MOS-sleep	FOSQ, JSS
Depression	BDI, HADS-D	BDI-II, HAMD17	BDI	—
Anxiety	HADS-A	Beck Anxiety Inv.	Beck anxiety inv.	—
Pain	Pain VAS, Pain NRS	BPI-mod short form	Pain VAS, McGill pain quest.	Pain VAS
Physical disability	FIQ, F-HAQ	FIQ, SDS	FIQ, MDHAQ	FIQ
Global/QOL	FIQ, CGI-C, PGI-C, SF-36	EQ-5D, FIQ, CGI-S, PGI-I, SF-36	FIQ, PGI-C, SF-36	FIQ, CGI-C, CGI-S, PGI-C, SF-36
Tenderness	MTPS-FIS	—	—	TPI

BDI: beck depression inventory, BPI: brief pain inventory, CGI-C: clinician global impression of change, CGI-S: clinician global impression of severity, EQ-5D: EUROQOL 5-D qUESTIONNAIRE, F-HAQ: fibromyalgia health assessment questionnaire, FIQ: fibromyalgia impact questionnaire, FIS: fibromyalgia intensity scale, FOSQ: Functional outcomes of sleep questionnaire, HADS: hospital anxiety and depression scale (-D denotes depression subscale, -A denotes anxiety subscale), HAMD17: 17-item hamilton rating scale for depression, Inv.: inventory, JSS: jenkins sleep scale, MAF: multidimensional assessment of fatigue, MASQ: multiple abilities self-report questionnaire, MDHAQ: multi-dimensional health assessment questionnaire, MFI: multidimensional fatigue inventory, MOS-sleep: medical outcomes studies sleep scale, MTPS: manual tender point survey, NRS: numeric rating scale, PGI-C: patient global impression of change, PGI-I: patient global impression of improvement, QOL: quality of life, QUEST.: questionnaire, SDS: sheehan disability scale, SF-36: short form health survey, TPI: tender point index, VAS: visual analogue scale.
